# Paper-Based SERS Platform for One-Step Screening of Tetracycline in Milk

**DOI:** 10.1038/s41598-019-54380-y

**Published:** 2019-11-29

**Authors:** Ana Marques, Bruno Veigas, Andreia Araújo, Beatriz Pagará, Pedro Viana Baptista, Hugo Águas, Rodrigo Martins, Elvira Fortunato

**Affiliations:** 1 i3N|CENIMAT, Departamento de Ciência dos Materiais, Faculdade de Ciências e Tecnologia, Universidade NOVA de Lisboa, Campus de Caparica, 2829-516 Caparica, Portugal; 2 UCIBIO, Departamento de Ciências da Vida, Faculdade de Ciências e Tecnologia, Universidade NOVA de Lisboa, Campus de Caparica, 2829-516 Caparica, Portugal; 3 INCM, Imprensa Nacional Casa da Moeda, Lisboa, Portugal

**Keywords:** Assay systems, Chemistry, Nanoscience and technology

## Abstract

Throughout the last decade, the expansion of food testing has been gradually moving towards ordinary high throughput screening methods performed on-site. The demand for point-of-care testing, able to distinguish molecular signatures with high accuracy, sensitivity and specificity has been significantly increasing. This new requirement relies on the on-site detection and monitorization of molecular signatures suitable for the surveillance of food production and processing. The widespread use of antibiotics has contributed to disease control of livestock but has also created problems for the dairy industry and consumers. Its therapeutic and subtherapeutic use has increased the risk of contamination in milk in enough concentrations to cause economic losses to the dairy industry and have a health impact in highly sensitive individuals. This study focuses on the development of a simple Surface-Enhanced Raman Spectroscopy (SERS) method for fast high throughput screening of tetracycline (TET) in milk. For this, we integrate a paper-based low-cost, fully recyclable and highly stable SERS platform, with a minimal sample preparation protocol. A two-microliter sample of milk solutions spiked with TET (from 0.01 to 1000 ppm) is dried on a silver nanoparticle coated cardboard substrate and measured via a Raman spectrophotometer. The SERS substrate showed to be extremely stable with a shelf life of several months. A global spectrum principal component analysis approach was used to test all the detected vibrational modes and their correlation with TET concentration. Peak intensity ratios (455 cm^−1^/1280 cm^−1^ and 874 cm^−1^/1397 cm^−1^) were found to be correlated with TET concentrations in milk, achieving a sensitivity as low as 0.1 ppm. Results indicate that this SERS method combined with portable Raman spectrometer is a potential tool that can be used on-site for the monitoring of TET residues and other antibiotics.

## Introduction

Nowadays, antibiotics are extensively used not only for the control of diseases but also for nutritional purposes of livestock. However, it’s massive use has created problems for the milk transformation industry and consumers^1,2^. The residues of antibiotics in milk can cause several problems to the final consumer such as, allergic reactions and development of antibiotic-resistance microorganisms, as well as being responsible for the disruption of Ca^2+^ metabolism which is harmful to the formation of teeth and bones in children. Less common effects are induced photosensitivity and increased hypersensitivity to light^1–5^. When antibiotics are used, dairymen should follow specific protocols to prevent the prevalence of antibiotic residues in human milk. Despite that, reports show that there is still presence of antibiotics in milk, above the recommended values^1,6^. Their relative stability to the pasteurization processes and the high quantities of milk required to dilute milk from treated quarters, can cause monetary losses and technological problems to the dairy industry. So, the use of monitoring tests by regulatory authorities and the dairy personnel have shown to drastically decrease the occurrence of antibiotic-adulterated milk^1,2^. Nowadays, the most common methods employed for antibiotics residues monitoring include microbial inhibition tests^7,8^, immunoassays^9,10^, and chemical-physical methods such as high-performance liquid chromatography^11,12^ or mass spectroscopy^13,14^. However, these methods have high operational costs, complex sample preparation, normally require a high quantity of reagents volume and specialized personnel and the samples analyzed per time unit are few^2,6^.

Nanoscale enabled technologies have been revolutionizing the routine laboratory detection schemes. These platforms may substitute the standard approaches used in molecular detection and characterization at centralized facilities. The main impact of this technology focus on miniaturization and increased sensitivity, allowing for low sample volume and decreased analysis time^15,16^. From these technologies, SERS has been gaining some attraction, since it can enhance Raman signals up to 14 orders of magnitude^17–19^. Recent advances in nanoscience catalyzed an explosion of possibilities regarding nanostructured substrates design and fabrication^15,16,20–23^. The development of improved SERS substrates together with portable Raman spectrometers is allowing this technique to spread into the point-of-need molecular detection and characterization scene^6,20^. One of the benefits of SERS is that it allows analyte’s detection without the need for any sample prep, with a high sensitivity.

The performance of the SERS technique depends on the choice of materials and nanostructures of the SERS-active substrate^15,19,20^. Recently, cellulose based substrates have been developed for several opto-electronic applications due to its unique set of advantages (e.g. 100% recyclable, flexible and low-cost)^21,24–26^. These exciting applications of paper-based materials for bio-detection, are today presenting several advantages over conventional substrates, due to their inexpensive and easy-to-process nature, achieving high Raman signal enhancements (EF ≈ 10^5^–10^7^) comparable with the commercial counterparts^16,27^. However, the adaptation of these substrates to large scale production is still difficult and complex, especially in the validation, reproducibility and standardization factors^21,28^. Once these issues are solved, it is expected that these platforms will expand from the conceptual demonstrations to real products.

Currently, the most common methods to fabricate these substrates have critical obstacles such as time-consuming complex patterning processes associated with high costs, which limits its extensive use in macroscopic scale systems^15,20,26^. Metal deposition by e-beam evaporation results in the direct arrangement of individual nanoparticles (NPs), with a good control of size and shape, without the need for any thermal treatment^21,29^. This one-step approach consists on the formation of Ag NPs *in situ* during the thermal evaporation of thin films of Ag deposited on substrates that are heated up to 150 °C^21^. This considerable low temperature is applicable to a broad range of low-cost and flexible substrate materials, that are not compatible with the conventional annealing de-wetting methods carried out at higher temperatures (300–500 °C)^21,30–32^. This process allows for a simple and inexpensive production of SERS substrates that are highly stable, homogeneous in terms of morphology and exhibit prominent Raman enhancement effects throughout the substrate^21^.

This work reports on highly efficient SERS platforms based on the AgNPs combined with an inexpensive, recyclable and widely used cardboard substrate, commonly used as disposable packaging material, previously fully described in a previous work from our group^21^.

In this work, we show the proof-of-concept of using this nanoplasmonic platform for the analysis of antibiotic residues in milk, demonstrating that this one-step thermal evaporation production method combined with an statistical analysis is able to exhibit a very strong SERS signal, with notable stability and shelf-life. This approach allows for a single-step detection and quantification of TET in milk without the need for any sample’s pre-treatment.

## Results and Discussion

### Substrate production and characterization

SERS substrates were produced on cardboard packaging by thermal deposition of a 6 nm Ag film. The cardboard is composed by a paper-based layer coated with evaporated aluminum. In contact with air, a thin layer of Al_x_O_y_ is formed which is essential for the growth of metallic NPs by the solid-state dewetting method. An optimization of SERS response to the Ag NPs of various shapes and sizes was systematically investigated elsewhere, in a previous work of some of the authors^21^. A electromagnetic simulation using the Mie theory was performed and helped determine that 6 nm Ag thin film, that renders 60 nm-sized Ag NPs gives the best SERS substrate for molecular detection^21^. This optimal local electric field enhancement is able to increase the Raman signal up to 10^6^ ^21^. To calculate this enhancement factor, it was used the area under the rhodamine 6G spectra’s peak at 1360 cm^−1^ of the SERS platform and of the cardboard without the AgNPs. This factor was calculated according to the literature^21^ using the expression I_SERS_ × C_SERS_/I_Raman_ × C_Raman_, where I_SERS_ and C_SERS_ is the area under the 1360 cm^−1^ peak and the concentration of rhodamine (10^−6^ M), respectively in the SERS platform and, I_Raman_ and C_Raman_ is obtained from the cardboard substrate, where C_Raman_ is 10^−3^ M. Reproducibility and stability analysis of the produced substrates was also assessed showing an intensity variation lower than 5% along the substrate area, with a shelf life higher than 6 months without loss in SERS performance^21,33^. This high shelf life is due to the lower oxidation of the Ag NPs in the presence of the aluminum layer in the cardboard structure. This metal layer acts as a sacrificial layer, providing anodic protection and preventing possible oxidation of the NPs. This is due to the higher standard reduction electrode potential of silver in comparison with aluminum – silver has a higher tendency to reduce and aluminum to oxidize^21^.

**Figure 1:**
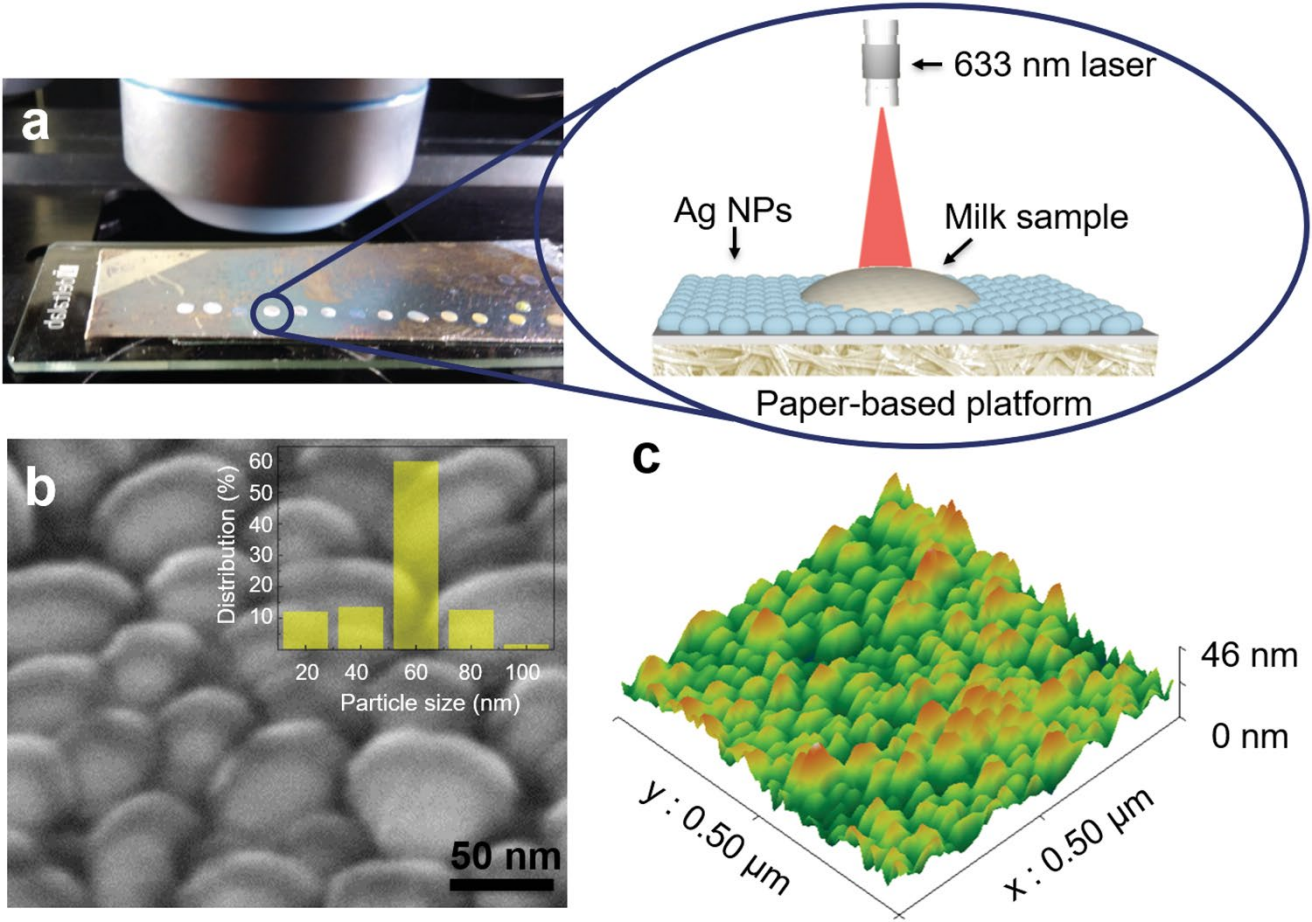
Production and characterization of cardboard-Ag NPs SERS substrates. (**a**) Optical photograph of the Ag NPs-cardboard platform with milk spiked TET measurement set up. The zoom in represents a schematic representation of a single measurement. (**b**) SEM image of the Ag NPs on top of the cardboard substrate. The histogram for the size particle distribution is presented in the inset image. (**c**) AFM image showing the cardboard substrate with Ag NPs.

Scanning electron microscopy and atomic force microscopy analyses of the cardboard substrates after the NPs deposition have been carried out (Fig. 1).

The SEM image of the produced Ag NPs (Fig. 1b), on top of the cardboard substrate, reveals a highly dense and uniform distribution of the nanostructures with an elongated spherical shape and an average diameter of 60 nm, as showed in the histogram. The uniformity and close proximity of the nanostructures, produced by thermal evaporation, contributes to the high reproducibility of SERS and to the Raman signal enhancement, due to the presence of hot spots (narrow gaps between neighbors NPs), that are excited by the surface plasmon coupling^34^. Additionally, AFM measurements of the NPs revealed an average height of ~46 nm, with typical in-plane ellipsoid axes ratios of ~1.3 (Fig. 1c).

### Implementation of plasmonic cardboard substrate as SERS device

**Figure 2:**
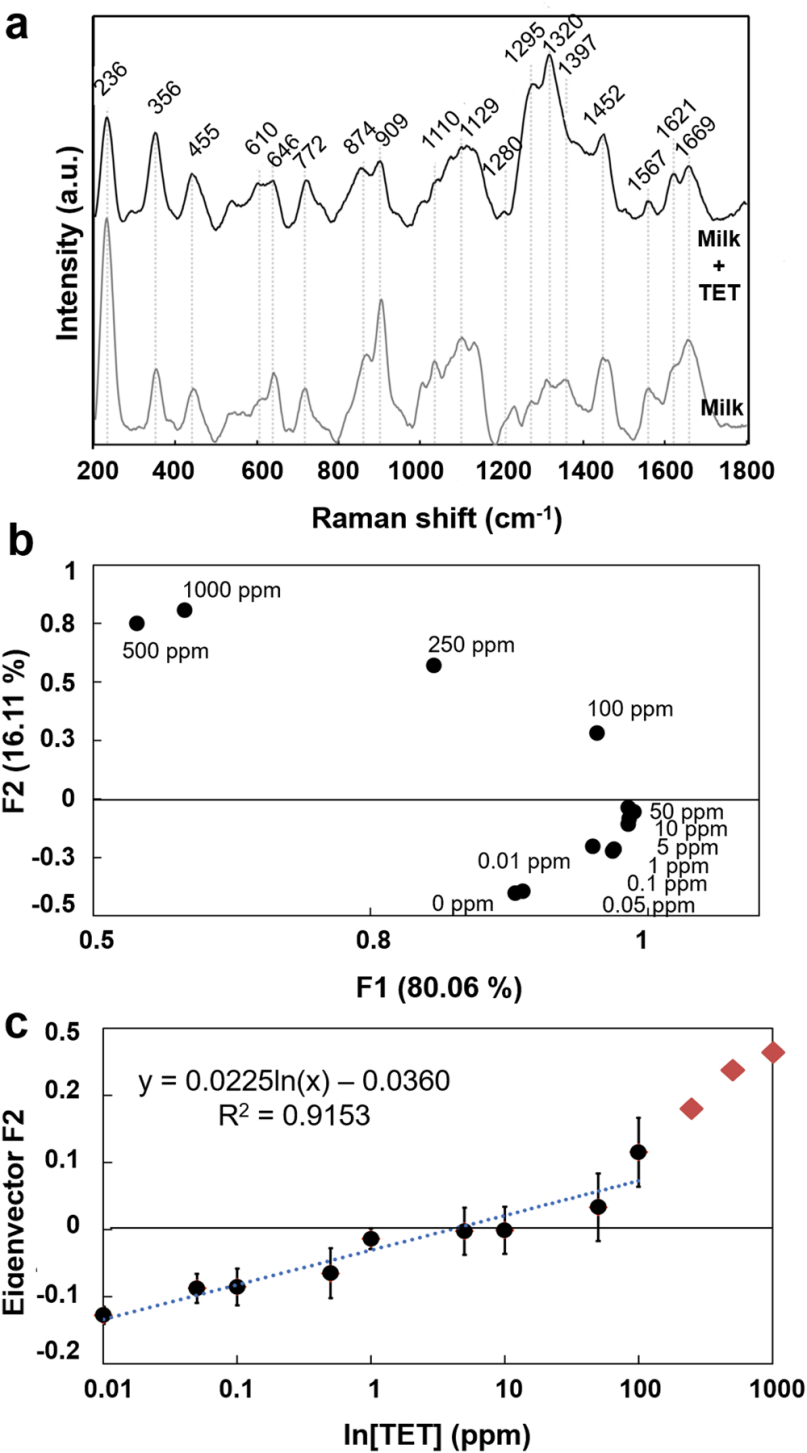
Raman spectra of TET spiked milk analysis (n = 3). (**a**) Raman spectra of milk (bottom) and 500 ppm TET spiked milk (top) showing its characteristic peaks. (**b**) Principal component analysis of TET detection and quantification in cardboard SERS substrate. The two eigenvectors F1 & F2 accounting for 96.12% of total variance in the data set. (**c**) Correlation between the eigenvector F2 variance and TET concentration. Each point corresponds to the mean value and error bars to the standard deviation of three independent measurements. A linear correlation (R^2^ = 0.9153) between the variance of the vector F2 and TET concentration is observed within the desired range (0–10 ppm).

To test the ability of reproducible detection and quantification of TET with this platform, SERS spectra of whole milk with added calibrated concentrations of TET were acquired. A two-microliter sample was deposited onto the SERS substrate and allowed to dry, after which four individual spectra were measured for each concentration. The measured SERS spectra of milk with and without TET (500 ppm) are shown in Fig. 2a. The SERS spectra of a few representative tested TET concentrations is presented in Supplementary Fig. S1.

Figure 2a shows the SERS spectra of milk and milk spiked with 500 ppm of TET. The characteristic peaks of milk and TET were only visible when the enhancement effect of the Ag colloid nanoparticles was used. The SERS spectra of TET in aqueous solution was recorded and is presented in Supplementary Fig. S2, showing TET characteristic Raman shifts. Additionally, to the characteristic milk peaks in Fig. 2a, a set of more pronounced peaks appear in the TET spiked milk at 1280 cm^−1^, 1322 cm^−1^ and 1621 cm^−1^. This evidence is in accordance with the TET characteristic peaks reported in the literature^6,35^ although with a small shift, due to the interaction of the TET with the milk matrix. The peak at 1280 cm^−1^ is correlated to bending modes of C-H in the positions 4, 4a, 5, 5a, O-H12 and amid-NH and to stretching modes of C10, 3-O, C-H7, 8, 9, C4a-C5 and amid-NC. The peak at 1322 cm^−1^ is assigned to ring breathing of stretching modes C6a-C7, C9-C10 and bending modes of O-H10, 12 and also to C-H4, 4a, 5, 5a. Finally, the peak at 1621 cm^−1^ is attributed to the stretching modes of C1,3-O, C2-C3 and amid-CO and to the breathing modes of amid-CO and amid-NH.

A principal component analysis (PCA) was performed to all the characteristic peaks (Fig. 2b), obtained in triplicates. The PCA showed that 96% of the variance was comprised in two eigenvectors. Most of the variance (80%) in all the analyzed characteristic peaks only showed a correlation to TET concentrations above 100 ppm. However, the second eigenvector corresponding to 16.1% of total variance showed a good correlation (R^2^ = 0.9153) for lower concentration of (<100 ppm) (Fig. 2c). While PCA is extremely powerful to access the correlation between spectral changes and analyte concentrations we envisioned a simplified measurement procedure that reduced the acquisition time while allowing to acquire more data points, thus circumventing one of the drawbacks of SERS-based detection approach (ensure sample representativeness).

Previous reports have used these characteristic peaks and peak ratios for the quantification of antibiotics in whole milk^6^. However, the PCA analysis showed that for lower analyte concentrations most of these peaks (present in the eigenvector F1 and representing 80% of the total variance) may not be the most appropriate approach for low quantification concentration window (see Supplementary Fig. S3). As such, in this approach, we analyzed all the possible combinations of peak ratios and their correlation to the concentration of the analyte (see Supplementary Fig. S4).

**Figure 3:**
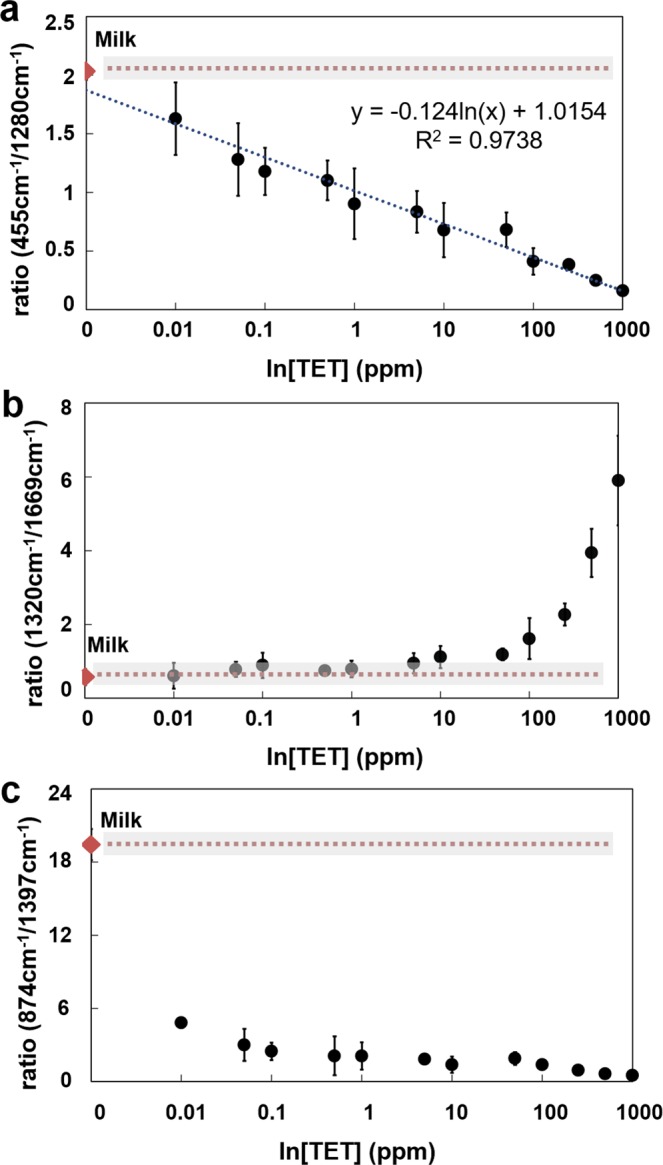
Dependence of SERS signal intensity ratio and TET concentration (n = 3). (**a**) Correlation between ratio 455 cm^−1^/1280 cm^−1^ versus analyte concentration in logarithmic scale. Linear correlation y = −0.124ln(x) + 1.0154; R^2^ = 0.9738). (**b**) Correlation between ratio 1320 cm^−1^/1669 cm^−1^ versus analyte concentration in logarithmic scale. (**c**) Correlation between ratio 874 cm^−1^/1397 cm^−1^ versus analyte concentration in logarithmic scale. Diamond marker shows the ratio values obtained for milk samples without TET and grey shade the error (standard deviation). Each point in the final calibration curve corresponded to the mean value and the error bars to the standard deviation of three independent measurements.

The characteristic milk peaks used in the showed ratios, at 455 cm^−1^, 1397 cm^−1^ and 1669 cm^−1^, although with small shifts, can be attribute to lactose, amino acids (such as glycine and l-valine) and to amide I, respectively^36,37^. The disappearance of the 1397 cm^−1^ peak in the milk/TET spectrum it is possibly due to the interaction of TET with the protein fraction of milk. Evidence of this interaction has been previously shown elsewhere, where it is described the adsorption capability of TET to milk^38^. From all peak and peak ratio intensities present in the full spectra the ratio 455 cm^−1^/1280 cm^−1^ (Fig. 3a) showed a linear correlation in a logarithmic scale (R^2^ = 0.97) for the entire tested range (0–1000 ppm). For this ratio, the limit of detection (LOD) was found to be 0.1 ppm. Another two peak combinations showed high correlation to analyte concentration (1320 cm^−1^/1669 cm^−1^ and 874 cm^−1^/1397 cm^−1^). The ratio 1320 cm^−1^/1669 cm^−1^ (Fig. 3b) is a clear representation of the correlation found in the PCA F1 vector analysis, with an LOD above 100 ppm of TET, well above the desired sensitivity needed for this application. Moreover, the ratio 874 cm^−1^/1397 cm^−1^ showed to have a LOD lower than 0.01 ppm of TET (Fig. 3c). The present work follows a label-free approach for the simple detection and quantification of TET residues in milk with high-throughput, with a LOD within the maximum value defined by government associations. The SERS platform here employed presents an EF within the state-of-the-art and it is comparable with other works for the detection of residues in food with a label-free approach^39^. Although some reports, based on SERS^40^ or fluorescence-based methods^41^ report lower LOD for the detection of TET, they comprise complex procedures for the fabrication of the sensing platforms. Nonetheless, even having a higher LOD our SERS platform and detection method is able to answer to the maximum value of TET residues in milk, as defined by the United States Food and Drug Administration^42^ (0.3 ppm) and by the European Union^43^ (0.1 ppm). Additionally, these results prove that a simple two peak measurement approach is reliable for the practical detection of TET residue in milk. Also, the use of peak ratios allows for a faster measurement protocol making it possible to sample up to 100 times more spots within the sample while maintaining a low measurement time.

## Conclusions

Since the first conceptual demonstration of SERS for food analysis, several reports have been published with several types of analytes. Herein we demonstrate the use of a new platform, with a highly simplified sample handling and data analysis protocol. We believe that this conceptual assay represents a valuable alternative to currently used methodologies for identification and quantification of analytes. Furthermore, high reproducibility and stability of this SERS substrate was also demonstrated. The reported high tunability of surface plasmon resonance combined with the stable and reproducible nature of these substrates demonstrate their effectiveness to be applied in large area substrate for SERS.

All characteristic vibrational modes of milk and TET spiked milk, obtained in triplicate, were analyzed via a global PCA-based analysis allowing to develop a peak ratio approach for the detection and quantification of tetracycline without any sample pretreatment. The simplification of the detection protocol allows for a higher number of measurements within the same sample to have a better statistical representation and correlation of the spectral data to the target analyte concentration. This approach showed a sensitivity bellow 0.1 ppm, which is the maximum residue limit set by the governmental authorities. These results serve as a foundation to further explore this method for other analytes and samples types. The development of stable SERS substrates together with the portable Raman spectrophotometers might radically change the way we perform analyte detection and quantification at point-of-need.

## Methods

### Preparation and characterization of SERS platform

Tetracycline hydrochloride (TET) and tetraethylrhodamine hydrochloride (rhodamine 6G) were purchased from Sigma-Aldrich and used as received without further purification. Distilled water was passed through a Millipore water system (ρ = 18.2 MΩ), which was used in all of the experiments. Commercially obtained ultra-high-temperature processing (UHT) semi-skimmed milk, from a Portuguese brand “Mimosa” was used through all the experiments. The cardboard substrate, with a thickness of 0.4 mm, was supplied by StoraEnso (Helsinki, Finland) and silver metal pieces with 99,99% purity, supplied by CERAC, Inc. (Milwaukee, Wisconsin, USA), where used for AgNPs production.

The SERS platform production protocol is fully described and characterized elsewhere^21^. The cardboard packaging substrates used in this study is composed by, pressed cellulose fibers, polymeric coatings and evaporated aluminum, with a thin native oxide (Al_x_O_y_) layer present on top of the aluminum layer. Ag NPs were obtained by the deposition of metal layers directly on the cardboard substrate (10 × 10 cm^2^) using an electron gun-assisted thermal evaporation technique, while the substrate was kept at 150 °C during the thermal evaporation. The deposition was carried out with a working pressure of 10^−5^ mbar and a deposition rate of 0.07 nm s^−1^ until the thickness of the Ag film reached 6 nm. The thicknesses and growth rates of the films were inferred by a calibrated quartz crystal detector.

The cardboard substrate surface after the NPs deposition was imaged by scanning electron microscopy with a Carl Zeiss AURIGA CrossBeam (FIB-SEM) workstation. The average height of the NPs was measured with an Asylum MFP3D atomic force microscope (AFM) in ac mode.

### SERS measurements and statistical analysis

Tetracycline was chosen as a model analyte to investigate the performance of the AgNPs-coated cardboard substrate for SERS detection in complex matrixes. The SERS substrates were prepared by dropping 2 μl of whole milk with added concentrations of TET with concentrations ranging from 0.01 to 1000 ppm onto the substrate. Samples were allowed to dry at room temperature (Fig. 1a).

Raman measurements were carried out with a Renishaw® inVia™ Qontor® confocal Raman microscope equipped with a Renishaw Centrus 2957T3 detector and a 633 nm laser operating at 50 mW. The spectra were recorded as an extended scan. The laser beam was focused with a 50× Olympus objective lens. All of the measurements were made with five scans of 10 s laser exposure. The 521 cm^−1^ peak of a silicon wafer was used between the different Raman sessions to calibrate the spectrograph for possible fluctuations of the Raman system.

Data is expressed as mean ± standard deviation from at least three independent experiments. Statistical analysis was performed using GraphPad Prism version 6.00 software. Principal Component Analysis was performed using XLStats 2014 software, to highlight the similarities and differences in the data. An orthogonal transformation was performed to convert a set of observations of possibly correlated variables into a set of values of linearly uncorrelated variables (principal components).

## Supplementary information

Supplementary Information

Dataset 1

## Data Availability

The dataset related with this manuscript is available on a separated excel file.
