# Supplementary material for: Paper-Based SERS Platform for One-Step Screening of Tetracycline in Milk
Source: Sci Rep. 2019 Nov 29;9:17922. doi: 10.1038/s41598-019-54380-y (PMC6884497; doi:10.1038/s41598-019-54380-y)
Supplement: Supplementary file 1 — Supplementary Information [file 41598_2019_54380_MOESM1_ESM.pdf]

Supplementary Information

Paper-Based SERS Platform for One-Step Screening of Tetracycline in Milk

Ana Marques[1†], Bruno Veigas[1,2,†], Andreia Araújo[1,3], Beatriz Pagará[1], Pedro Viana Baptista[2], Hugo Águas[1], Rodrigo Martins[1] and Elvira Fortunato[1,*]

[1] i3N|CENIMAT, Departamento de Ciência dos Materiais, Faculdade de Ciências e Tecnologia, Universidade NOVA de Lisboa, Campus de Caparica, 2829-516 Caparica, Portugal; accm@campus.fct.unl.pt (A.M.); bmrveigas@gmail.com (B.V.); andreiajoiaraujo@hotmail.com (A.A.); pmvb@fct.unl.pt (P.V.B.); hma@fct.unl.pt (H.A.); emf@fct.unl.pt (E.F.)

[2] UCIBIO, Departamento de Ciências da Vida, Faculdade de Ciências e Tecnologia, Universidade NOVA de Lisboa, Campus de Caparica, 2829-516 Caparica, Portugal

[3] INCM, Imprensa Nacional Casa da Moeda, Lisboa, Portugal

* Correspondence: emf@fct.unl.pt (E.F.)

† Equal contribution.

Keywords: SERS; tetracycline; food testing; paper-based; single-step screening

The SERS spectra of a few representative TET concentration in milk was also recorded with a 633 nm laser, and it is presented in Figure S1.

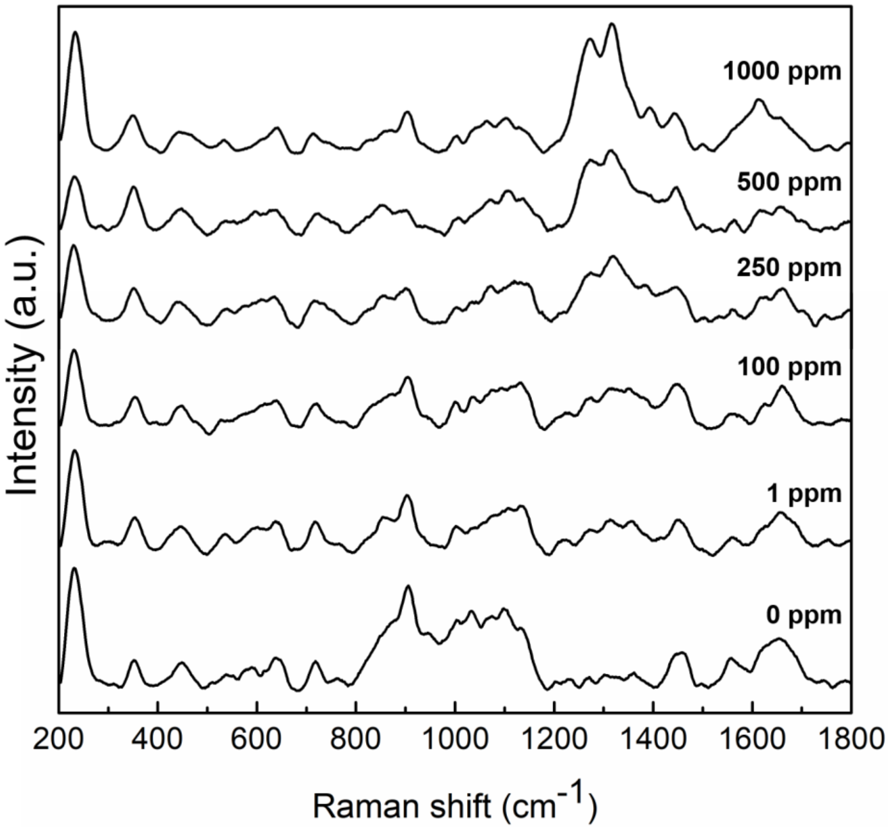

Figure S1: SERS spectra of a few representative concentrations of TET spiked milk.

The SERS spectra of 500 ppm tetracycline (TET) aqueous solution, was recorded with a 633 nm laser. The characteristic peaks of TET are represented in Figure S2.

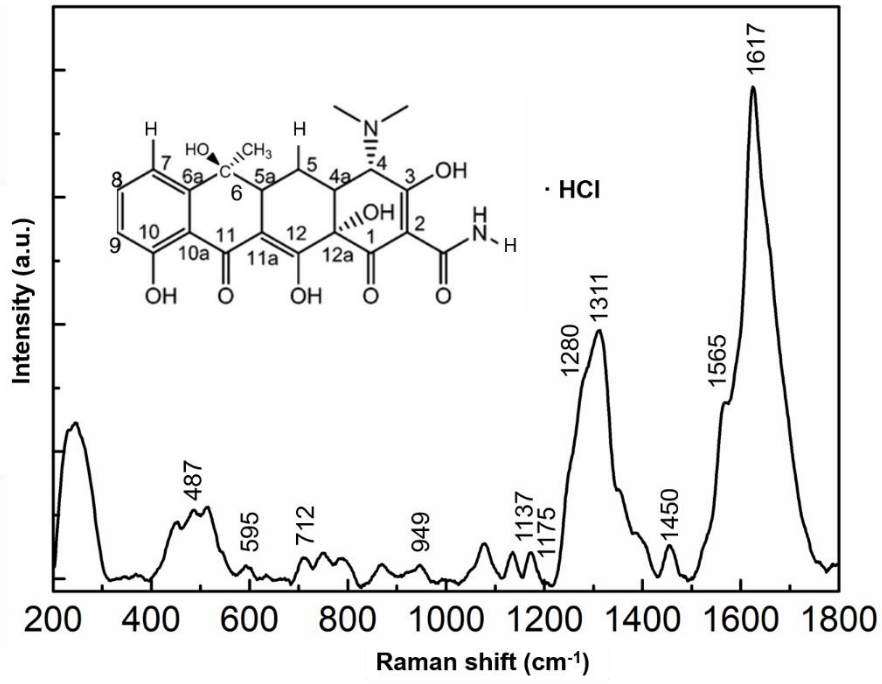

Figure S2: SERS spectra of 500 ppm TET in aqueous solution showing TET characteristic peaks. These peaks are reported in the literature although with small deviations. The inset shows the TET hydrochloride molecule with positional number of carbon atoms adapted from Mitscher, L. A. The Chemistry of the Tetracycline Antibiotics; Marcel Dekker: New York, 1978.

A principal component analysis (PCA) was performed to TET characteristic peaks. The PCA showed that 96% of the variance was comprised in two eigenvectors (F1 and F2). Most of the variance (80%) in all the analyzed characteristic peaks only showed a correlation to TET concentrations above 100 ppm (Figure S3).

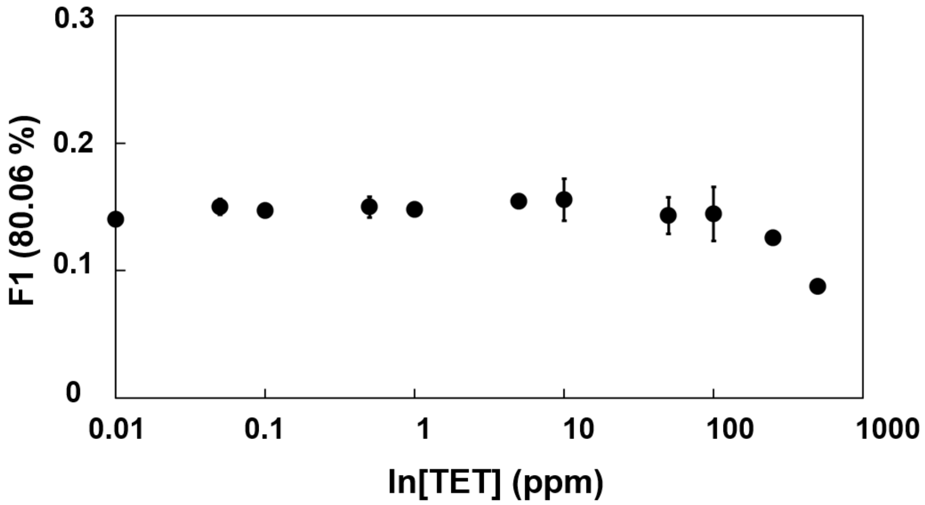

Figure S3: Correlation between the eigenvector F1 variance and TET concentration. Each point corresponds to the mean value and error bars to the standard deviation of three independent measurements. A linear correlation between the variance of the vector F2 and TET concentration is observed for concentration values above 100 ppm.

For lower analyte concentrations most of these peaks (present in the eigenvector F1 and representing 80% of the total variance) are not be the most appropriate approach for low quantification concentration window.

In this study **we** analyzed all the possible combinations of peak ratios and their correlation to the concentration of the analyte (Figure S4).

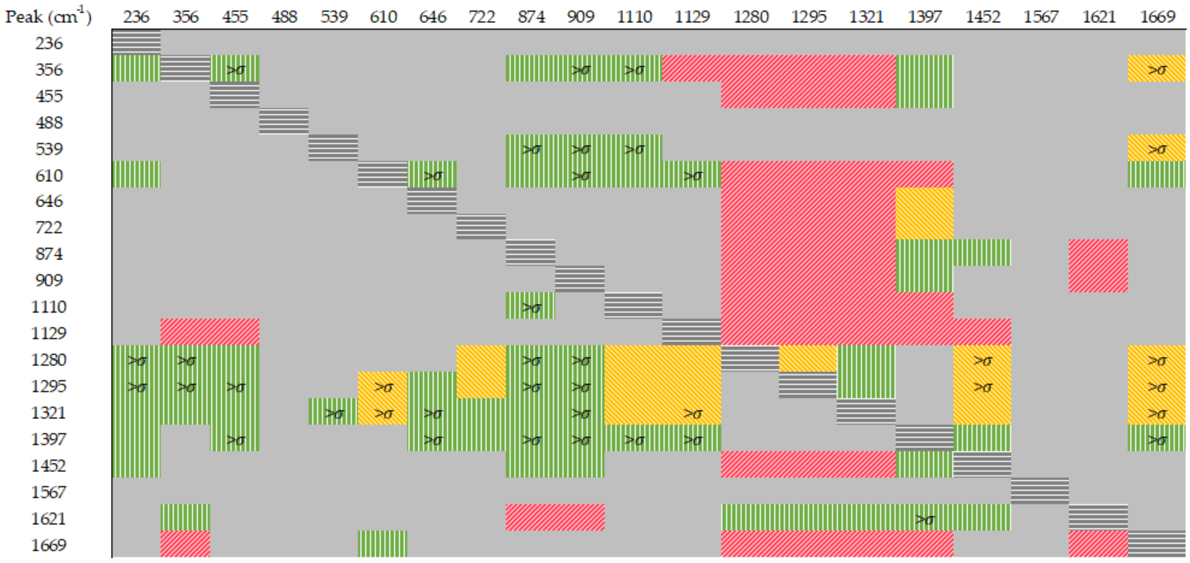

Figure S4: Combinations of peak ratios and their correlation to the concentration of the analyte. **Grey** (▤): No correlation between ratio intensity and analyte concentration was found; **Green** (▥): Correlation between ratio intensity and analyte concentration was found above 100 ppm; **Yellow** (▧): Correlation between ratio intensity and analyte concentration was found above 1 ppm; **Red** (▨): Correlation between ratio intensity and analyte concentration was found above 0.01 ppm. Ratios with high standard deviation values between measurements are marked with (>**σ**).
